# Involvement of a Bacterial Microcompartment in the Metabolism of Fucose and Rhamnose by *Clostridium phytofermentans*

**DOI:** 10.1371/journal.pone.0054337

**Published:** 2013-01-28

**Authors:** Elsa Petit, W. Greg LaTouf, Maddalena V. Coppi, Thomas A. Warnick, Devin Currie, Igor Romashko, Supriya Deshpande, Kelly Haas, Jesús G. Alvelo-Maurosa, Colin Wardman, Danny J. Schnell, Susan B. Leschine, Jeffrey L. Blanchard

**Affiliations:** 1 Department of Microbiology, University of Massachusetts, Amherst, Massachusetts, United States of America; 2 Department of Biochemistry and Molecular Biology, University of Massachusetts, Amherst, Massachusetts, United States of America; 3 Department of Veterinary and Animal Sciences, University of Massachusetts, Amherst, Massachusetts, United States of America; 4 Department of Biology, University of Massachusetts, Amherst, Massachusetts, United States of America; University of Florida, United States of America

## Abstract

**Background:**

*Clostridium phytofermentans*, an anaerobic soil bacterium, can directly convert plant biomass into biofuels. The genome of *C. phytofermentans* contains three loci with genes encoding shell proteins of bacterial microcompartments (BMC), organelles composed entirely of proteins.

**Methodology and Principal Findings:**

One of the BMC loci has homology to a BMC-encoding locus implicated in the conversion of fucose to propanol and propionate in a human gut commensal, *Roseburia inulinivorans*. We hypothesized that it had a similar role in *C. phytofermentans*. When *C. phytofermentans* was grown on fucose, the major products identified were ethanol, propanol and propionate. Transmission electron microscopy of fucose- and rhamnose-grown cultures revealed polyhedral structures, presumably BMCs. Microarray analysis indicated that during growth on fucose, operons coding for the BMC locus, fucose dissimilatory enzymes, and an ATP-binding cassette transporter became the dominant transcripts. These data are consistent with fucose fermentation producing a 1,2-propanediol intermediate that is further metabolized in the microcompartment encoded in the BMC locus. Growth on another deoxyhexose sugar, rhamnose, resulted in the expression of the same BMC locus and similar fermentation products. However, a different set of dissimilatory enzymes and transport system genes were induced. Quite surprisingly, growth on fucose or rhamnose also led to the expression of a diverse array of complex plant polysaccharide-degrading enzymes.

**Conclusions/Significance:**

Based on physiological, genomic, and microarray analyses, we propose a model for the fermentation of fucose and rhamnose in *C. phytofermentans* that includes enzymes encoded in the same BMC locus. Comparative genomic analysis suggests that this BMC may be present in other clostridial species.

## Introduction

*Clostridium phytofermentans* was isolated from forest soil near the Quabbin Reservoir in Massachusetts, U.S.A [1]. Phylogenetically, it is a member of Cluster XIVa of the low-GC-content Gram-positive bacteria, which includes human gut commensals [2]. *C. phytofermentans* ferments all major pentose and hexose components of lignocellulose yielding ethanol and hydrogen as the major fermentation products [1]. Because of its ability to convert plant biomass directly to ethanol, *C. phytofermentans* is being developed as a catalyst for commercial biofuel production [3].

Three loci encoding bacterial microcompartments (BMCs) are present within the *C. phytofermentans* genome (Petit et al., In preparation). BMCs (also called bacterial microcompartments, carboxysomes, metabolosomes, polyhedral bodies, and protein microcompartments) are relatively large, cytoplasmic, macromolecular complexes (100 to 150 nm in cross section) that are bound by a crystalline layer of shell proteins [4], [5] and contain metabolic enzymes both within and in association with the polyhedral shell [6], [7]. The first BMCs to be identified were the carboxysomes of the cyanobacteria [8], which contain enzymes involved in the fixation of carbon dioxide, including ribulose-1,5-bisphosphate carboxylase/oxygenase (RuBisCO) [9], [10] and carbonic anhydrase. Carboxysomes are thought to concentrate carbon dioxide in close proximity to the RuBisCO complex, thereby enhancing autotrophic carbon dioxide fixation at atmospheric levels of carbon dioxide [11].

Homologs of the cyanobacterial carboxysome shell proteins have also been identified in enteric bacteria [6], [12]. In *Salmonella enterica*, BMC shell protein genes are found within two unusually large operons, *pdu* (21 genes) and *eut* (17 genes), that encode enzymes involved in the catabolism of 1,2-propanediol and ethanolamine, respectively [7], [12]. Sequestration of these enzymes within the polyhedral shell is thought to protect the cell from toxic metabolic intermediates and increase the efficiency of 1,2-propanediol and ethanolamine metabolism [11], [13], [14].

New BMC loci, many of as yet unknown function, are continuously being identified as bacterial genomes are sequenced [4], [15]. Comparative genomic analysis indicated that there are seven functionally distinct families of BMC loci distributed among more than 40 genera of bacteria [16]. BMC loci are present in the genomes of several clostridia, including the human gut microbe, *Roseburia inulinivorans*, which belongs to the same clostridial phylogenetic clade (XIVa) as *C. phytofermentans* [17]. Transcriptional analysis performed during growth of *R. inulinivorans* on fucose suggested that a BMC was involved in the synthesis and metabolism of 1,2-propanediol, an intermediate in the conversion of fucose to propionate and propanol [17].

In *E. coli*, *S. enterica*, and other bacteria, 1,2-propanediol is produced during the metabolism of fucose and rhamnose [18], [19]. Fucose and rhamnose are naturally occurring deoxyhexose sugars (sometimes called methyl-pentoses) found in the glycans on the surfaces of mammalian and bacterial cells and in the membranes and cell walls of many plant and insect species. In *E. coli* and presumably *S. enterica*, fucose and rhamnose are metabolized via parallel pathways that converge at the production of lactaldehyde and dihydroxyacetone phosphate (DHAP) [20]. DHAP can then feed into the glycolytic pathway where it can be converted to a variety of fermentation products, whereas lactaldehyde is converted to 1,2-propanediol and secreted. In *S. enterica*, the 1,2-propanediol can be taken back up and converted to propanol and propionate by enzymes found within the *pdu* microcompartment locus [21].

In the present study, we report genomic, transcriptional, and physiological evidence indicating that a BMC-encoding locus containing many genes homologous to those found within the BMC locus of *R. inulinivorans*, is involved in the metabolism of fucose and rhamnose by *C. phytofermentans*.

## Materials and Methods

### Sequence analysis

The genome of *C. phytofermentans* ISDg is complete (Accession number NC_010001) and was used as the basis of the sequence analysis. Related protein sequences were identified using blastp (http://blast.ncbi.nlm.nih.gov/Blast.cgi). To confirm that sequences were absent from the genome and that negative outcomes were not the result of missing protein predictions, sequencing errors or the formation of a recent pseudogene, the *C. phytofermentans* genome nucleotide sequence was also searched using tblastn (http://blast.ncbi.nlm.nih.gov/Blast.cgi). Each protein sequence was also searched against the domain database of National Center for Biotechnology Information (NCBI) to further infer potential functions (www.ncbi.nlm.nih.gov/Structure/cdd/cdd.shtml). The complete and draft bacterial genome databases (downloaded on July 14, 2011) were searched for homologs of the *C. phytofermentans* BMC locus using a local version (2.2.10) of NCBI tools.

### Culturing conditions

*C. phytofermentans* was cultured in a modified form of an anaerobic medium [1] containing the following (g/l): yeast extract, 6.0; urea, 2.1; KH_2_PO_4_, 4.0; Na_2_HPO_4_, 6.5; trisodium citrate dihydrate, 3.0; L-cysteine hydrochloride monohydrate, 2.0; and resazurin, 1; with pH adjusted to 7.0 using KOH. This medium was supplemented with 0.3% (wt/vol) of the specific substrate (glucose, fucose or rhamnose) added as a filter-sterilized solution to the sterile medium. Triplicate liquid cultures were incubated at 30°C under anaerobic conditions (100% N_2_) as described by Hungate [22]. Growth was determined spectrophotometrically by monitoring changes in optical density at 660 nm.

### Determination of fermentation products

Non-gaseous fermentation products were determined by HPLC and GC. Acetate, ethanol, formate and lactate concentrations in culture supernatants were measured using a BioRad Aminex HPX 87H 300×7.8 mm column at 55°C with 0.005 M H_2_SO_4_ as the mobile phase and a flow rate of 0.60 ml/min, in a Hitachi model L-7100 HPLC unit equipped with a Sonntek Refractive Index Detector. Concentrations of 1-propanol and ethanol were measured by GC, using a Shimadzu GC 2014 with a Flame Ionization Detector and a Restek stabilwax-DA 30 m×0.25 mm ID column (film thickness 0.25 µm). The carrier gas was helium at a flow rate of 1.5 ml/min. Injector and detector temperatures were both 200°C and the column temperature began at 70°C for 2 min, ramped to 175°C at 20°C/min, and was held at 175°C for 2 min.

### Electron Microscopy

*C. phytofermentans* cells from fucose- and rhamnose -grown cultures were grown until mid-exponential phase, fixed for 1 hr at room temperature with 2.5% glutaraldehyde, 0.03% picric acid, and 0.5% NaCl in 0.05 M cacodylate buffer and washed three times in 0.05 M cacodylate buffer and 0.5% NaCl. Gel pellet formation was performed by adding 3% agarose, incubated in ice for 15 min followed by post-fixation with 1% osmium tetraoxide, and 1.5% K ferrocyanide in 0.05 M cacodylate buffer for 2 hrs. The gel pellets were washed with water followed by *en bloc* staining with 2% uranyl acetate for 1 hr, followed by three additional water washes and subsequent dehydration. Samples were embedded in 26% Epon-812, 15.5% Araldite-502, 57% Dodecenylsuccinic anhydride and epoxy accelerator DMP-30 1.5% and polymerized at 60°C overnight. Thin sections were cut at 70 nm using a Reicher-Jung Ultracut E microtome, stained with lead citrate, and observed in a JEOL 100× transmission electron microscope at 100 kv.

### BMC purification

BMCs were partially purified from fucose-grown cultures of *C. phytofermentans* under anaerobic conditions. Cells were harvested from two mid-exponential phase, 500 ml cultures (OD at 660 nm between 0.3 and 0.4) by centrifugation at 10,000×g for 10 min at 4°C. To reduce flagellar contamination of the BMC preparations, cells were resuspended in 200 ml of TE buffer (10 mM Tris/HCl pH 8.0, 1 mM EDTA) and sheared in a Waring blender for 15 sec. Sheared cells were harvested by centrifugation at 10,000×g for 20 min at 4°C and resuspended in 10 ml of Cellytic B2x buffer (Sigma-Aldrich) supplemented with 0.2 mg/ml lysozyme, 50 units/ml of Benzonase nuclease (Sigma-Aldrich), and 1 ml of protease inhibitor cocktail for bacterial cells (Sigma-Aldrich). Following a 30-min incubation at room temperature with continuous shaking, unlysed cells and cell debris were pelleted by centrifugation at 12,000×g for 20 min and the supernatant was reserved. The resulting pellet was further treated with 10 ml of Cellytic B2x, supplemented as described above, for 20 min at room temperature with continuous shaking. Following clarification by centrifugation at 12,000×g for 20 min, this supernatant was pooled with the supernatant derived from the first Cellytic B2x buffer treatment and subjected to high speed centrifugation at 50,000×g for 90 min at 4°C to pellet organelles. The resulting BMC-containing pellet was resuspended in 10 ml TEMP buffer [23]. Unresuspended debris were pelleted by centrifugation at 12,000×g for 10 min. BMCs were enriched from this clarified suspension by concentrating it to a final volume of 1 ml in a stirred ultrafiltration cell (Pall Corporation) that was pressurized with N_2_ gas and equipped with a 300K omega (polyethersulfone) membrane (Pall Corporation).

Purifed BMCs were negatively stained with 1% uranyl acetate as described by Pohlschröder et al. [24]. Thin sections and negatively stained preparations were examined using a JEOL 100S transmission electron microscope.

### RNA isolation for microarray analysis

RNA was isolated from two replicates of each of the three types of cultures (glucose, rhamnose, and fucose). RNA was purified from mid-exponential phase cultures at 27 hrs for glucose and 62 hrs for fucose and rhamnose. Samples were flash-frozen by immersion in liquid nitrogen. The cells were collected by centrifugation for 5 min at 8,000 rpm at 4°C. Harvested cells were re-suspended in 100 µl in TE buffer pH 8 (EMD Chemicals) containing 2 mg/ml lysozyme (Sigma-Aldrich) and incubated at 37°C for 40 min. Total RNA was isolated using RNeasy RNA purification kit (QIAGEN) according to the manufacturer's instructions. Contaminating DNA in total RNA preparations was removed with RNase-free DNase I (QIAGEN). The RNA concentration was determined by absorbance at 260/280 nm using a Nanodrop spectrophotometer.

### Microarray design

A *C. phytofermentans* Affymetrix microarray was custom- designed for the measurement of the expression level of all open reading frames, estimation of the 5′ and 3′ untranslated regions of mRNA, operon determination, and discrimination between alternative gene models (differing primarily in the selection of the start codon). Putative protein coding sequences were identified using both GeneMark [25] and Glimmer [26], and the union of these two predictions was used to design the array. For transcriptional analysis, each coding sequence (CDS) was represented by eleven 24-mer probes. Standard Affymetrix array design protocols were followed to ensure each probe was unique to minimize cross hybridization. If two CDS differed only in their N-terminal region, the smaller of the two proteins was used for transcript analysis, but the extended region was also represented by probes in order to define the actual N-terminus. Remaining probes were used to map expression in intergenic regions. These probes represented both DNA strands and were tiled with a 1-nucleotide gap. The array design was implemented on a 49-5241 format Affymetrix GeneChip with 11 µm features.

### Microarray processing

cDNA synthesis, array hybridization and imaging were performed at the Genomic Core Facility at the University of Massachusetts Medical Center. Ten µg total RNA from each sample was used as template to synthesize labeled cDNAs using Affymetrix GeneChip DNA Labeling Reagent Kits. The labeled cDNA samples were hybridized on the arrays according to Affymetrix guidelines. The hybridized arrays were scanned with a GeneChip Scanner 3000. The resulting raw spot image data files were processed into pivot, quality report, and normalized probe intensity files using Microarray Suite version 5.0 (MAS 5.0). In addition, expression values were calculated using the Custom Array Analysis Software (CAAS) package (http://www.sourceforge.net/projects/caas-microarray/) that implements the Robust Multichip Average method [27]. The individual microarray files (Accession numbers GSM333247 to GSM333252) and the normalized gene summary values for the complete data set (Accession number GSE13194) have been deposited in Gene Expression Omnibus (GEO) database at NCBI (www.ncbi.nlm.nih.gov/geo/).

The quality of the microarray data sets were analyzed using probe-level modeling procedures provided by the affyPLM package [28] in BioConductor [29]. No image artifacts due to array manufacturing or processing were observed. Microarray backgrounds were within the typical 20–100 average background values for Affymetrix GeneChip. In summary, all quality control checks indicated that the RNA purification, cDNA synthesis, labeling and hybridization procedures adapted for use in *C. phytofermentans* resulted in high quality data.

All microarray data reported in the text and figures represent the average of expression values derived from two independent RNA preparations from duplicate cultures.

### Reverse transcription and quantitative PCR

Cultures were grown and harvested for RNA during mid-exponential phase as described above. RNA was extracted using a detergent-based method [30]. cDNA was produced using the QIAGEN Quantitect Reverse Transcription kit as per manufacturer's directions with a 10 min incubation for the gDNA Wipeout step and a 30 min incubation for the reverse transcription step. PCR amplification of the GAPDH gene was performed to confirm cDNA production and controls were run without reverse transcriptase to check for gDNA contamination. qPCR standards were made by PCR amplification of purified genomic DNA with appropriate primers (File S1), followed gel extraction (QIAGEN QIAquick Gel Extraction Kit) and ethanol precipitation. Two additional washes were added to the gel extraction protocol to improve the efficiency of the extraction. qPCR was performed using Bio-Rad's iTaq Universal SYBR Green Supermix. Each reaction consisted of 7.8 µL MilliQ water, 10 µL iTaq Supermix, 0.6 µL of each primer, and 1 µL of cDNA or standard. Standards, experimental samples, and blanks were run in triplicate on the same plate for 40 cycles (15 seconds at 95°C, 30 seconds at 58°C, 30 seconds at 72°C). A 5 min 95°C hot start was used. After the 40 cycles, the reactions were completed with 7 min at 72°C, followed by 5 min at 30°C, and finally a melting curve from 60°C to 95°C. Samples were cooled to 21°C after the melting curve. In addition to melting curves, all samples and blanks were run on 1.5% agarose gels at 120 v for 30 min and photographed under UV light to probe for false positives caused by primer-dimers.

## Results

### A BMC locus with a putative role in fucose metabolism in the genome of *C. phytofermentans*

Analysis of the *C. phytofermentans* genome revealed a 14-gene, BMC-encoding locus (Table 1). All of the genes within this locus are homologous to genes encoded in the BMC locus of *R. inulinivorans* (67–90% similar, Table 1). The *R. inulinivorans* BMC operon was identified during microarray analysis comparing gene expression during growth on fucose and glucose [17]. During growth of *R. inulinivorans* on fucose, the BMC operon was expressed at high levels and the major fermentation products were propionate, propanol and butyrate, consistent with the occurrence of 1,2-propanediol metabolism within the BMC [17].

**Table 1:** Composition and differential expression of the *C. phytofermentans* BMC locus.

*Clostri-dium phytoferm-entans* protein	*Roseburia inulinivorans* homolog	*Salmonella enterica* functional equivalent^b^	Characterized homolog^c^		Proposed protein function	Fold change in expression relative to glucose	
	(% simi-larity)^a^	(%simi-larity)^a^	Organism	(% similarity)^a^		Fucose	Rhamnose
Cphy_1174	ABC25539 (77)	PduCDE (nd)	*Clostridium butyricum*	Glycerol dehydratase with 1,2-propanediol dehydratase activity DhaBI (68)	Propanediol dehydratase	43.6	32.4
Cphy_1175	ABC25540 (76)	PduGH (nd)	*C. butyricum*	Glycerol dehydratase activator DhaB2 (56)	Propanediol dehydratase activator	57.5	38.8
Cphy_1176	ABC25524 (75)	PduU (58)	*-*	-	BMC shell protein (PF00936)	60.6	38.9
Cphy_1177	ABC25526 (83)	nd	*Methano-caldococcus jannaschii*	Fuculose aldolase FucA (57)	Fuculose and rhamnulose phosphate aldolase	86.1	62.2
Cphy_1178	ABC25528 (83)	PduP (62.6)	-	-	Propionaldehyde dehydrogenase	86.6	59.1
Cphy_1179	ABC25529 (84)	PduQ (nd)	*Oenococcus oeni*	Alcohol dehydrogenase with propanol dehydrogenase activity (37)	Propanol dehydrogenase	72.7	44.9
Cphy_1180	ABC25530 (67)	PduA (51)	*-*	-	BMC shell protein (PF00936)	74.7	50.2
Cphy_1181	ABC25532 (77)	PduK (53)	*-*	-	BMC shell protein (PF00936)	80.6	55.5
Cphy_1182	ABC25531 (90)	PduA (90)	*-*	-	BMC shell protein (PF00936)	73.7	47.4
Cphy_1183	ABC25534 (76)	PduL (63)	*-*	-	Phosphate propanoyl transferase	49.4	32.7
Cphy_1184	ABC25535 (83)	PduN (60)	*-*	-	EutN_CcmL shell protein (PF03319)	37.2	22.5
Cphy_1185	ABC25536 (67)	PduS (58)	*-*	-	Propanediol oxidoreductase	31.7	20.6
Cphy_1186	ABC25537 (78)	PduT (66.3)	*-*	-	BMC shell protein (PF00936)	25.6	15.7
Cphy_1187	ABC25538 (69)	PocR (nd)	*Escherichia coli*	Glucitol operon repressor SrlR (52)	Transcriptional regulator	2.1	1.8

a *All percent similarity values are calculated from pairwise global alignments of protein sequences generated using the algorithm of Needleman and Wunsch* [*45*] *, with the exception of those for Cphy_1178, 1181, and 1182. Only a segment of these three genes was homologous to designated proteins. Percent similarity values are reported for alignment of the entire homolog with a segment of the C. phytofermentans gene product (amino acids 7–184, 1–98 and 73–161 for Cphy_1178, Cphy_1181 and Cphy1182, respectively).*

b *Pdu (propanediol utilization) nomenclature based on that of S. enterica* [*37*] *.*

c *When significant amino acid similarity with an S. enterica protein (>35%) was not detected (n.d.), we attempted to identify the most closely related characterized homolog. References for characterized homologs are as follows: Clostridium butyricum B-12 independent glycerol dehydratase and activator* [*31*] *,* [*32*] *, M. jannaschii fuculose-phosphate aldolase* [*46*] *, O. oeni alcohol dehydrogenase* [*33*] *and E. coli glucitol operon repressor SrlR* [*47*] *.*

Several of the genes within the BMC loci of *R. inulinivorans* and *C. phytofermentans* are similar to genes found within the well-characterized 1,2-propanediol utilization (*pdu*) operon of *S. enterica*, which is involved in the conversion of 1,2-propanediol produced from rhamnose and fucose fermentation to propanol and propionate [7]. These include homologs of genes encoding BMC shell proteins (*pduU*-, *pduA*-, *pduK*-, *pduN*- and *pduT*-like) as well as genes encoding enzymes catalyzing the conversion of propionaldehyde to propionate (*pduP*-like and *pduL*-like) (Table 1). However, there are also important differences between the two clostridial BMC loci and the *S. enterica pdu* operon. One major difference is that the two clostridial loci lack homologs of genes that encode the B_12_-dependent 1,2-propanediol dehydratase (*pduCDE*) and its reactivation factors (*pduGH*). Instead, the BMC loci of *R. inulinivorans* and *C. phytofermentans* contain homologs of a *Clostridium butyricum* diol dehydratase with propanediol dehydratase activity and its associated activator protein (Table 1, [17]). The dehydratase of *C. butyricum* is not related to that of *S. enterica*, and is a single subunit enzyme that utilizes S-adenosylmethionine as a cofactor [31], [32]. The putative propanol dehydrogenase encoded within the BMC loci of *R. inulinivorans* and *C. phytofermentans* is likewise not related to that of *S. enterica* (PduQ) and appears to be a zinc-dependent alcohol dehydrogenase belonging to the medium chain dehydrogenase/reductase (MDR) superfamily [33], [34] (Table 1). Similarly, the transcriptional regulator, encoded within the *R. inulinivorans* and *C. phytofermentans* loci, belongs to the DeoR regulator family, and is not related to the transcriptional regulator of the *S. enterica pdu* operon, PocR (Table 1) [35], [36]. Finally, the BMC loci of *R. inulinivorans* and *C. phytofermentans* include a gene encoding an enzyme with homology to fuculose-phosphate aldolases, which is proposed to be involved in the production of lactaldehyde and DHAP (Table 1 and detailed further below).

As indicated in Table 1, comparison of the *C. phytofermentans* and the *R. inulinivorans* BMC clusters reveals a significant degree of overall similarity between the two loci, despite a few rearrangements. The major difference between the two loci is the absence of *pduV*- and *pduO*-like genes in the *C. phytofermentans* locus. The function of PduV is not currently known [37]. *In S. enterica*, PduO and PduS are involved in the synthesis of the adenosylcobalamin cofactor of the 1,2-propanediol dehydratase from vitamin B_12_ [38]. Because the *C. phytofermentans* and *R. inulinivorans* loci encode a type of dehydratase that is B_12_-independent, the genes involved in the synthesis of this cofactor may be dispensable or have alternate functions. The *pduS*-like gene encoded in the *R. inulinivorans* and *C. phytofermentans* BMC clusters, has been proposed to function as a 1,2-propanediol oxidoreductase [17].

A search of all complete and partial bacterial genomic sequences revealed that this BMC locus is present in other clostridial species. Two other Clostridium cluster XIVa human commensals, *Ruminococcus gnavus* and *Ruminococcus obeum*, share a similar BMC, but the majority of the cluster XIVa clostridia sequenced as part of the human gut microbe project do not. Many members of Clostridium Cluster I, including *Clostridium botulinum*, *Clostridium novyi*, *Clostridium beijerinckii* and *Clostridium tetani*, also possess this type of BMC.

### *C. phytofermentans* grown on fucose and rhamnose produces propanol and polyhedral microcompartments

**Figure 1:**
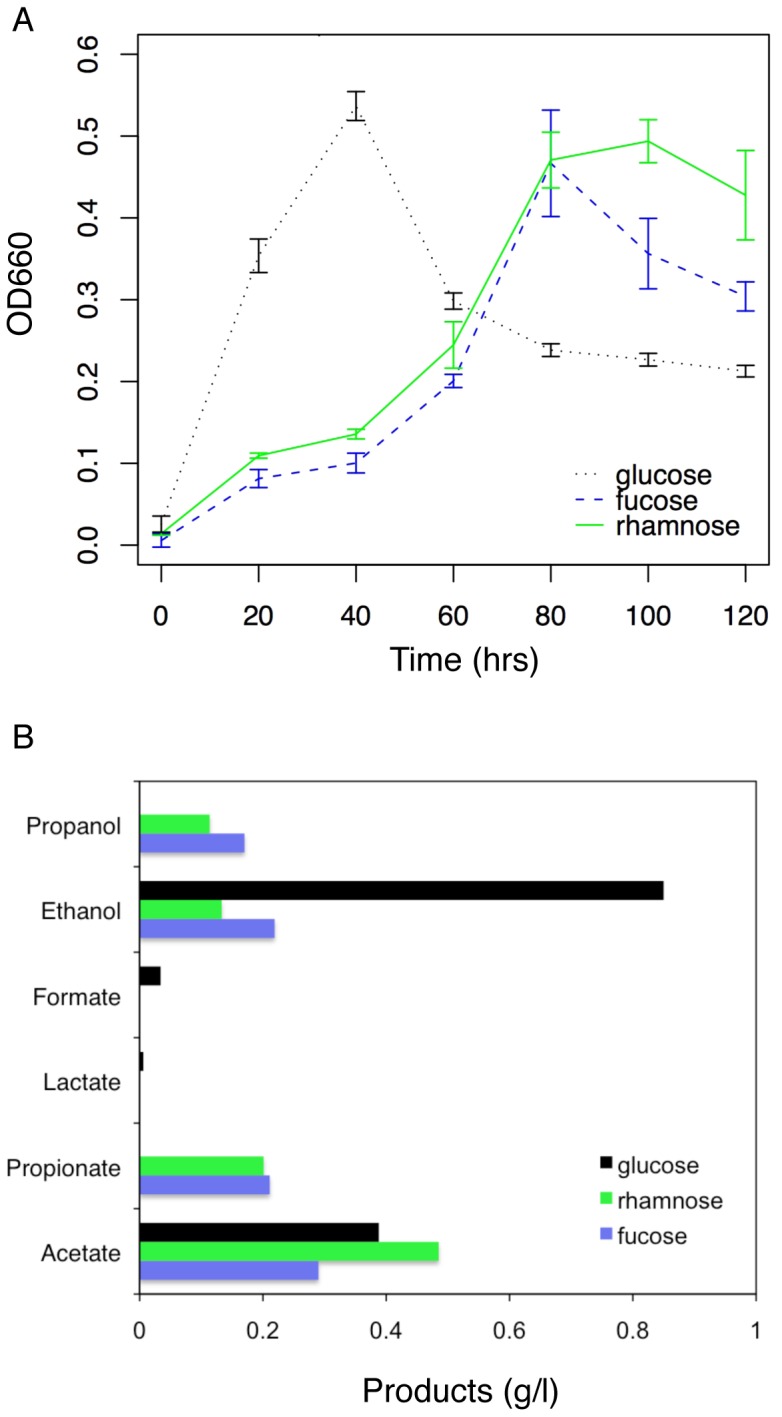
Growth and products produced by *C. phytofermentans* cultured on glucose, fucose or rhamnose. (A) Growth data are the average +/− standard deviations of three independent cultures. (B) Fermentation products were measured at 62 hrs on fucose and rhamnose and at 27 hrs on glucose and represent averages of measurements from the two cultures, which were used for RNA preparation.

Based on the genomic evidence described above, we hypothesized that the BMC locus participates in fucose and rhamnose metabolism in *C. phytofermentans*. To evaluate this hypothesis, *C. phytofermentans* was cultured in media containing glucose, fucose or rhamnose as the primary carbon source, and fermentation product formation and synthesis of BMCs were monitored. *C. phytofermentans* was capable of growth on both fucose and rhamnose, albeit at a slower rate than on glucose (Fig. 1). The cells were subcultured twice on their respective substrates prior to the growth measurements, however a longer lag phase exists during growth on fucose and rhamnose relative to glucose. During growth on glucose, *C. phytofermentans* produced ethanol as the major fermentation product, with acetate and lactate as minor products (Fig. 1). In contrast, the primary fermentation products during growth on fucose and rhamnose were acetate, lactate, ethanol, propionate, and propanol. Formation of these fermentation products is consistent with the metabolism of fucose and rhamnose via the pathway detailed further below. Briefly, fucose and rhamnose are converted to lactaldehyde and DHAP. Production of ethanol, acetate, and lactate would result from entrance of DHAP into the glycolytic pathway, whereas production of propionate and propanol could be explained by further metabolism of lactaldehyde by the enzymes found within the BMC.

**Figure 2:**
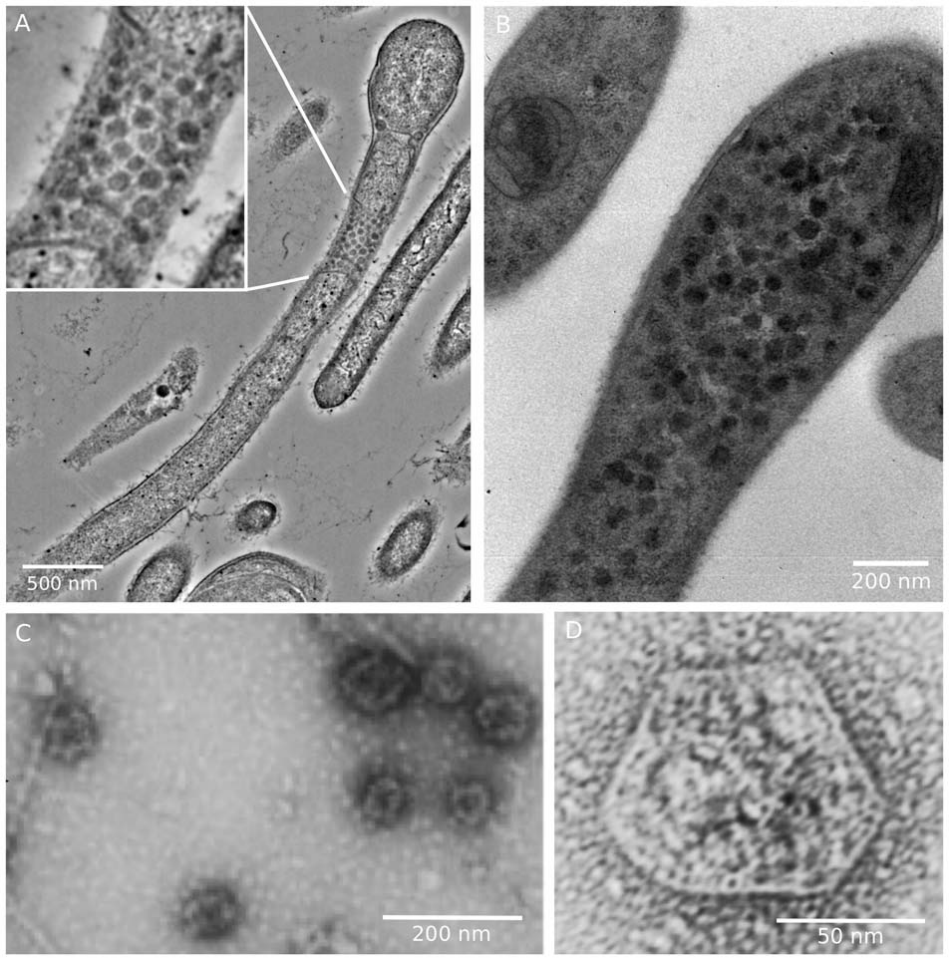
Transmission electron micrographs of *C. phytofermentans* BMCs. Thin section of cells cultured on fucose (A) and rhamnose (B) showing a cell with multiple polyhedral structures. (C) Flagellar contamination of negatively stained BMC preparation from mid-exponential phase cells cultured on fucose, showing flagella and BMCs. (D) BMC from mid-exponential phase cells cultured on fucose that were sheared of flagella.

Polyhedral structures, presumably BMCs, were visible by transmission electron microscopy of thin sections of *C. phytofermentans* cells growing on fucose and rhamnose (Fig. 2). It was possible to partially purify BMCs from mid-exponential phase fucose-grown cultures of *C. phytofermentans* (Fig. 2). Flagella were sheared from cells prior to cell lysis to avoid contamination of BMC preparations (Fig. 2). The BMC preparations, size-fractionated and concentrated by ultrafiltration, contained polyhedral structures, approximately 100 nm in cross section, that were not regular icosahedrons (Fig. 2), similar to purified Pdu and Eut microcompartments [5].

### Transcriptional response to growth on fucose or rhamnose includes the BMC locus and enzymes involved in the uptake and dissimilation of fucose and rhamnose and the degradation of complex polysaccharides

**Figure 3:**
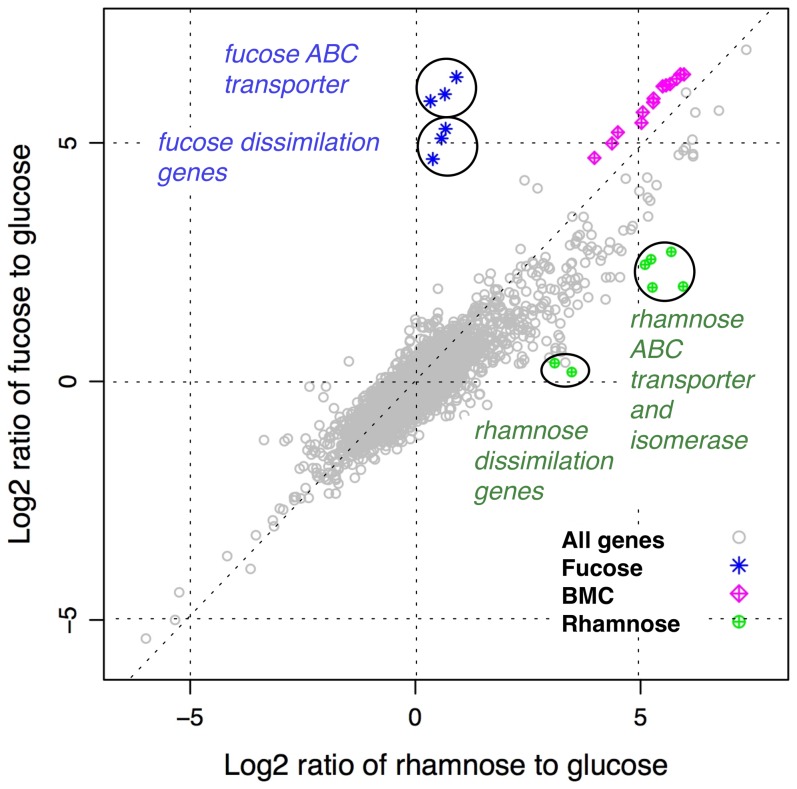
Scatterplot of gene expression on fucose and rhamnose relative to glucose. Expression values were calculated from the average of two rhamnose or fucose measurements divided by the average of two glucose measurements.

To gain further insight into the genes involved in the metabolism of rhamnose and fucose by *C. phytofermentans*, microarray analyses comparing global gene expression during growth on fucose or rhamnose to gene expression during growth on glucose were performed. The ratio of gene expression during growth on fucose relative to glucose was highly correlated with the ratio of gene expression during growth on rhamnose relative to glucose (Multiple R-Squared: 0.7785, p-value: <2.2e-16, Fig. 3). Therefore, with the exception of a very small subset of fucose- and rhamnose-related genes, the transcriptional response to the two sugars was nearly identical.

**Figure 4:**
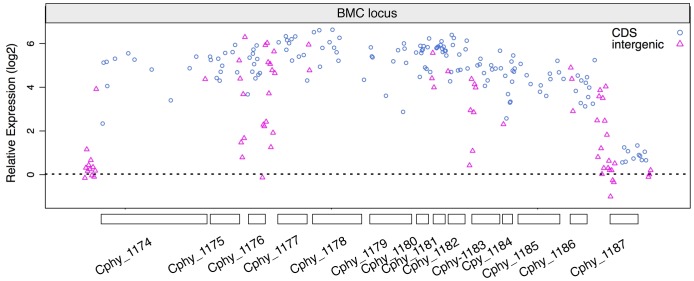
Expression values of individual probes within the BMC locus. Expression values were calculated from the average of two rhamnose measurements divided by the average of two glucose measurements. Each coding sequence is represented by eleven 24-mer probes. The intergenic regions have a variable numbers of probes depending on the length of the region.

**Figure 5:**
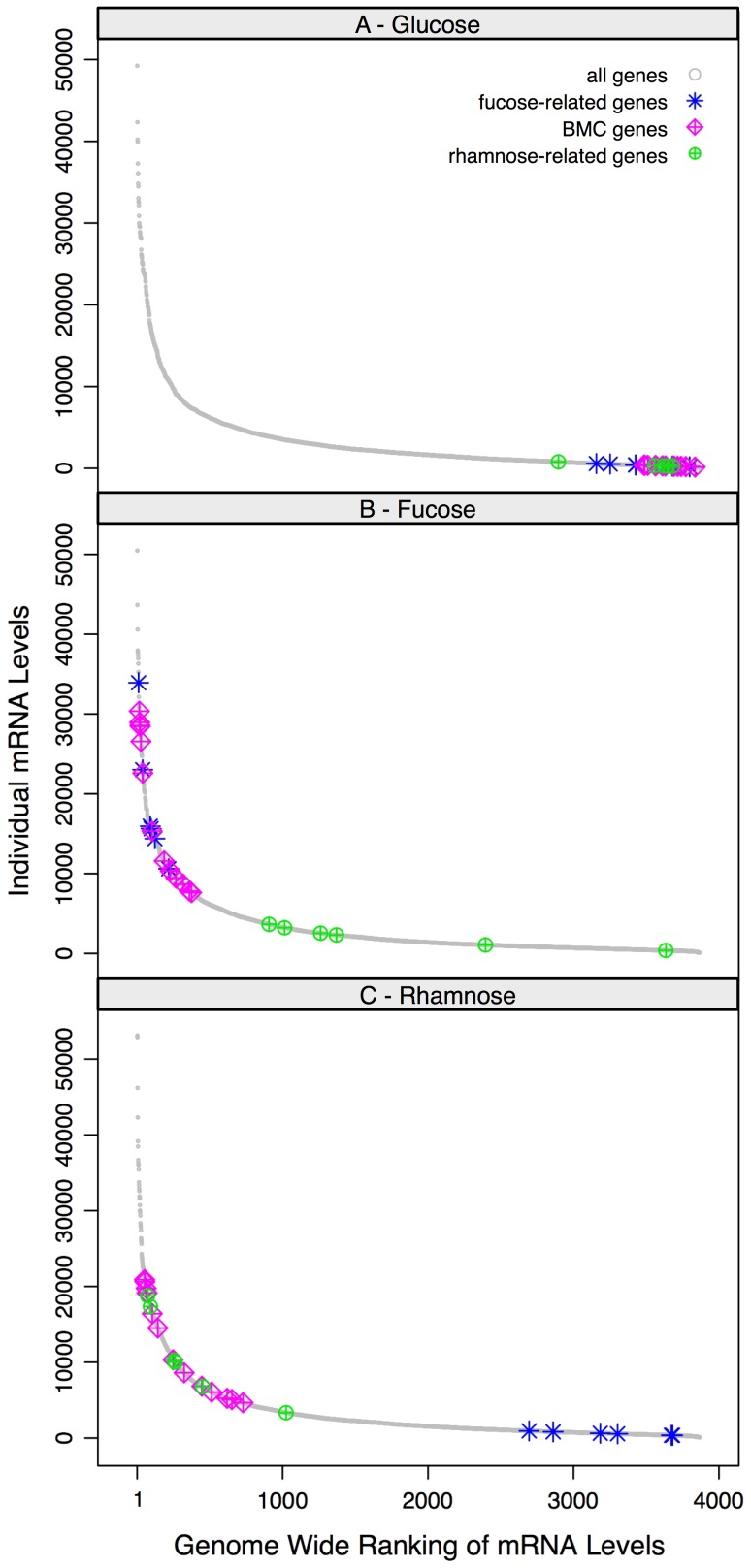
Rank abundance of mRNA expression levels. The expression levels of the BMC locus and genes involved in fucose and rhamnose transport and metabolism (fucose- and rhamnose-related, respectively) are plotted as a function of their genome-wide mRNA ranking level. The #1 ranked gene is the highest expressed protein-coding gene in the genome. Expression values are the average of two rhamnose, fucose or glucose measurements.

Expression of the genes within the BMC locus increased dramatically, 15–87 fold, during growth on fucose and rhamnose relative to glucose, with the exception of the transcriptional regulator that increased only twofold (Table 1). These data suggest that the BMC locus comprises two major, independently-regulated transcripts: a monocistronic transcription unit consisting of the transcriptional regulator gene (Cphy_1187) and a long BMC operon extending from the BMC shell-like gene (Cphy_1186) to the putative 1,2-propanediol dehydratase gene (Cphy_1174). Examination of the probe map of the BMC-locus supported this hypothesis (Fig. 4). The majority of probes within the intergenic regions between Cphy_1186 and Cphy_1174 were differentially expressed at levels similar to probes found within the flanking coding sequences (Fig. 4). During growth on fucose and rhamnose, the transcripts of the genes within the BMC operon were among the most abundant in the genome and fell within the 99^th^ percentile (Fig. 5). In contrast, during growth on glucose, the BMC operon was essentially shut off. These data are consistent with the BMC operon playing a role in the metabolism of fucose and rhamnose.

Analysis of the microarray data also lead to the identification of two fucose-related loci that were among the highest expressed in the genome (within the 99^th^ percentile) during growth on fucose and effectively shut off during growth on glucose and rhamnose (Table 2, Fig. 5). Examination of the probe maps of these loci indicated that they are both likely to constitute operons (data not shown). One of the putative fucose-related operons (Cphy_2010–2012) encodes a monosaccharide-importing ATP-binding cassette (ABC) transporter, likely to mediate fucose uptake. Both the ATP-binding (Cphy_2010) and permease (Cphy_2011) subunits of the transporter contain domains that are characteristic of hexose and pentose-importing ABC transporters; i.e. two similar ATP-binding domains (cd03215 and cd03216) in Cphy_2011 and a permease (cd06579) in Cphy_2012. The solute-binding subunit (Cphy_2012) contains a domain (cd01536) found in the periplasmic sugar-binding domain of uncharacterized ABC-type transport systems. A similar ABC transporter may also be involved in fucose uptake in *R. inulinivorans*; a fragment of an ABC-transporter solute binding protein with 60% similarity to Cphy_2012 was one of the most up-regulated transcripts during growth on fucose [17]. These results contrast with fucose uptake in *E. coli*, which is mediated by a single subunit fucose/proton symporter [39]. The second fucose-related operon (Cphy_3153–3155) encodes homologs of three enzymes involved in the conversion of fucose to fuculose-phosphate: a fucose maturotase, a fucose isomerase, and a fuculose kinase (Table 2).

**Table 2:** Description and relative expression values from microarray experiments of *C. phytofermentans* genes predicted to participate in the uptake and conversion of fucose and rhamnose to fuculose- and rhamnulose- phosphate, respectively.

*C. phytofermentans* protein	Predicted protein functions	Closest characterized functional equivalent^a^		Fold change in expression relative to glucose	
		Organism	Protein (% similarity)	Fucose	Rhamnose
**Fucose dissimilation**					
Cphy_3153	Fucose mutarotase	*Escherichia coli*	FucU (67)	25.5	1.4
Cphy_3154	Fuculose kinase	*Bacteroides thetaitaomicron*	FucK (60)	34.5	1.5
Cphy_3155	Fucose isomerase	*Caldicellulosiruptor saccharolyticus*	FucI (73.1)	39.5	1.6
**Fucose transport**					
Cphy_2010	Fucose ABC transport system, ATPase subunit	*E. coli*	FucP (n.d.)	58.4	1.3
Cphy_2011	Fucose ABC transport system, permease subunit	*E. coli*	FucP (n.d.)	82.3	1.9
Cphy_2012	Fucose ABC transport system,periplasmic solute binding protein	*E. coli*	FucP (n.d.)	64.9	1.6
**Rhamnose dissimilation**					
Cphy_1146	Rhamnulose kinase	*E. coli*	RhaB (56)	1.3	8.5
Cphy_1147	Rhamnose isomerase	*E. coli*	RhaA (70)	1.3	11.1
Cphy_1148	Transcriptional regulator of rhamnose metabolism	*E. coli*	RhaR (40)	0.8	2.7
Cphy_1149	Rhamnose mutarotase	*E. coli*	RhaM (72)	1.1	2.0
**Rhamnose transport**					
Cphy_0580	Rhamnose ABC transport system, ATPase component	*Rhizobium leguminosarum*	RhaT (62)	8.1	34.9
Cphy_0581	Rhamnose ABC transport system, permease subunit	*R. leguminosarum*	RhaP (57)	9.3	37.6
Cphy_0582	Rhamnose ABC transport system, permease subunit	*R. leguminosarum*	RhaQ (57)	10.8	51.5
Cphy_0583	Rhamnose ABC transport system, periplasmic solute-binding subunit	*R. leguminosarum*	RhaS (53)	10.7	62.4
Cphy_0584	Isomerase	n.i.	n.i.	7.0	38.3

a *All percent similarity values were derived from pairwise global alignments of protein sequences generated using the algorithm of Needleman and Wunsch* [*45*] *. n.d. indicates that significant amino acid similarity with the functional equivalent (>35%) was not detected. n.i. indicates that a characterized functional equivalent was not identified. References for characterized functional equivalents are as follows: E. coli fucose mutarotase FucU* [*48*] *, B. thetaitaomicron fuculose kinase* [*49*] *, Caldicellulosiruptor saccharolyticus fucose isomerase* [*50*] *, E. coli fucose transporter* [*39*] *and rhamnose dissimilation enzymes* [*41*] *,* [*51*] *and R. leguminosarum rhamnose transporter* [*40*] *.*

Two rhamnose-related loci, which were differentially up-regulated during growth on rhamnose were also identified (Table 2, Fig. 3 and 5). The most highly up-regulated locus (Cphy_0580-0584, transcript abundance within the 99^th^ percentile) was a single operon (probe map data not shown) encoding a monosaccharide-importing ABC transporter (Cphy_0580-0583) with a high degree of similarity to the rhamnose transporter of *Rhizobium leguminosarum* [40] and an isomerase-related protein of unknown function (Cphy_0584). Although expression of this putative operon was highest on rhamnose, it was also significantly up-regulated during growth on fucose (Table 2 and Fig. 3 and 5).

The second highly up-regulated rhamnose-related locus (Cphy_1146–1147) also appeared to be transcribed as an operon (probe data not shown) and encoded two enzymes involved in the conversion of rhamnose to rhamnulose-phosphate - a rhamnulose kinase and a rhamnose isomerase. An adjacent operon (Cphy_1148–1149) encoded the third enzyme required for rhamnose dissimilation, a rhamnose mutarotase, as well as an AraC family transcriptional regulator with moderate similarity to RhaR, the rhamnose transcriptional regulator of *E. coli* [41]. All of the rhamnose dissimilation enzymes displayed a high degree of similarity to those of *E. coli* (Table 2).

Thus, when *C. phytofermentans* was grown on fucose or rhamnose, the major transcripts, representing roughly 3% of the total RNA were the BMC and the sugar dissimilation and transport genes listed in Table 2.

To test whether the microarray results were in some way anomalous, quantitative PCR experiments were run on four selected genes, fucose isomerase, rhamnose isomerase, 1,2-propanediol dehydratase and a BMC shell protein postulated from genome sequence and microarray analysis to be involved in fucose and rhamnose metabolism (Table 3). The qPCR results on all four genes corroborated the sign and magnitude of the expression changes observed in the microarray results in Tables 1 and 2.

**Table 3:** Differential expression during growth on fucose or rhamnose relative to glucose as measured by qPCR of selected genes predicted to be involved in fucose and rhamnose metabolism.

	*C. phytofermentans* gene			
	Cphy_1147	Cphy_3155	Cphy_1184	Cphy_1174
Substrate	Rhamnose isomerase	Fucose isomerase	BMC shell protein	1,2-propanediol dehydratase
fucose rep1	1.0	19.6	38.7	58.4
fucose rep2	1.5	16.8	51.9	52.6
rhamnose rep1	55.4	1.1	91.7	132.2
rhamnose rep2	59.7	1.1	106.3	110.3

The genome of *C. phytofermentans* encodes multiple enzymes that could catalyze the release of fucose and rhamnose from oligosaccharides including three putative alpha-L-fucosidases (Cphy_2190, Cphy_3023, and Cphy_3028) and five rhamnogalacturonases (glycoside hydrolase family 28, Cphy_1711, Cphy_2567, Cphy_2736, Cphy_3217, Cphy_3310). Surprisingly, these predicted fucosidases and rhamnogalacturonases were constitutively expressed on all three sugars. Another surprising observation was the induction of expression of a variety of enzymes involved in the degradation of complex plant and fungal polymers that have no obvious value for *C. phytofermentans* during growth on either fucose or rhamnose. These included cellulases, chitinases, mannanases and other polysaccharide-degrading enzymes (Table 4).

**Table 4:** Differential expression of carbohydrate-active enzymes during growth on fucose and rhamnose relative to glucose.

			Fold change in expression relative to glucose	
Gene	Predicted protein function a	CAZy domain^a^	Fucose	Rhamnose
Cphy1800	Chitinase	GH18	33.4	71.1
Cphy1799	Chitinase	GH18	26.3	71.7
Cphy3368	Cellulase	GH48	22.1	47.4
Cphy3367	Cellulase	GH9	15.3	28.0
Cphy1163	Cellulase	GH5	8.2	11.9
Cphy2919	Pectate lyase	PL9	6.0	6.5
Cphy1888	Pectate lyase	PL9	2.4	6.4
Cphy1071	Beta-mannanase	GH26	5.5	11.5
Cphy2105	Xylanase	GH11	3.8	8.5

a As described in http://www.cazy.org/.

## Discussion

**Figure 6:**
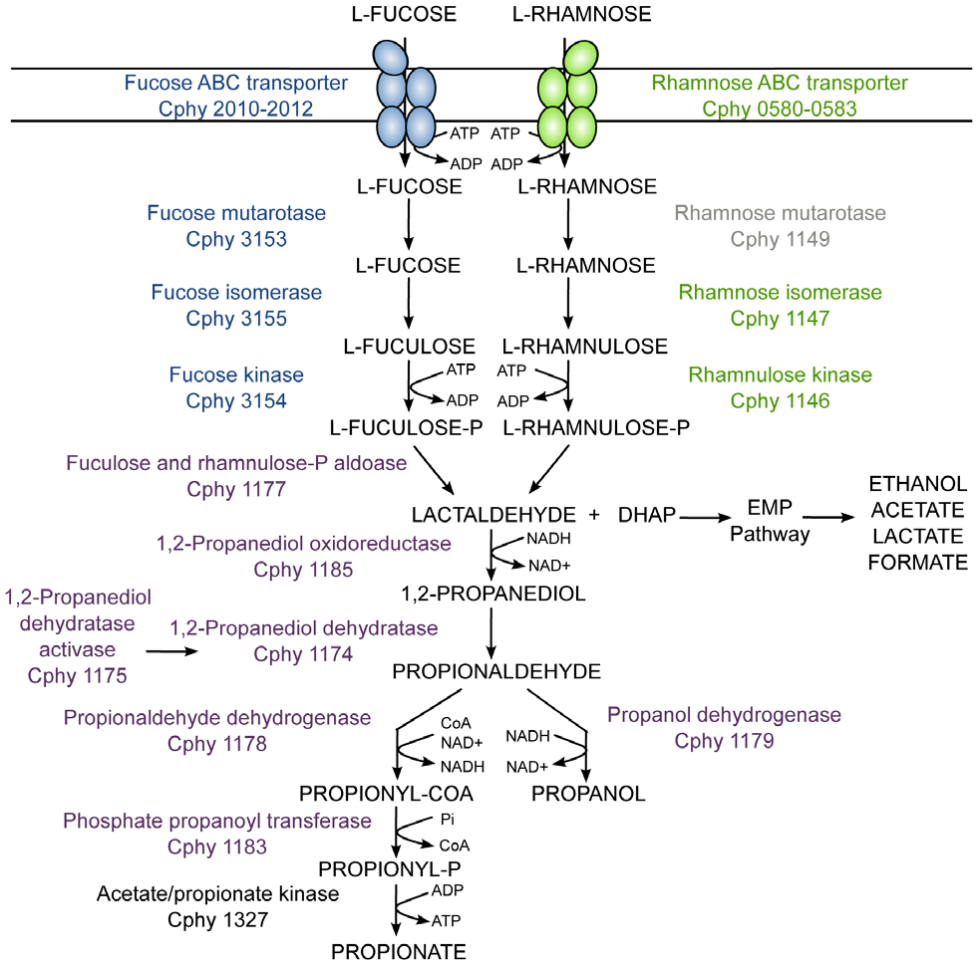
A model of *C. phytofermentans* fucose and rhamnose fermentation pathways. All gene functions are based on sequence homology and expression data. Gene names in blue, green and purple are respectively highly expressed on fucose, rhamnose and both. The genes names in purple are also part of the BMC locus. The rhamnose mutarotase is shaded gray, because there is only a small increase in its expression level in our experiments. The Embden-Meyerhof pathway is designated EMP.

Fucose and rhamnose are naturally occurring deoxyhexose sugars found in the cell membranes and walls of many plant species. Analysis of the *C. phytofermentans* genome suggested that *C. phytofermentans* should be capable of metabolizing both sugars to a variety of fermentation products via pathways partly encased in a BMC. *C. phytofermentans* was found to grow on both fucose and rhamnose, forming a variety of fermentation products including propanol, propionate, ethanol, lactate, and acetate. Structures resembling BMCs were observed in cells cultured on fucose. The combination of these results with microarray data comparing global gene expression during growth on fucose, rhamnose and glucose enabled the construction of a model for fucose and rhamnose catabolism by *C. phytofermentans* (Fig. 6). In this model, fucose and rhamnose are imported by specific ABC transporters. The fucose and rhamnose ABC transporter systems are of the CUT2-type, and these transport systems are commonly involved in monosaccharide transport [42]. The intracellular monosaccharides are metabolized by their respective mutarotases, kinases and isomerases resulting in fuculose- and rhamnulose-phosphate which are converted to lactaldehyde and DHAP by an aldolase encoded within the BMC locus. DHAP can enter the glycolytic pathway and be converted to multiple fermentation products including ethanol, acetic acid and lactate. Lactaldehyde is metabolized within the BMC and may be converted to 1,2-propanediol by a PduS-like propanediol-oxidoreductase. 1,2-propanediol is proposed to be converted to propionaldehyde by an S-adenosylmethione-dependent 1,2-propanediol dehydratase, based on similarity to the *Clostridium butyricum* B-12-independent glycerol dehydratase [31]. This enzyme is a member of the “radical SAM” or “AdoMet radical” superfamily [43]. The glycerol dehydratase is activated by the glycerol dehydratase-activating enzyme and requires AdoMet and strictly anoxic conditions [31]. *C. phytofermentans* has a homologous activating enzyme (Cphy_1175) that is adjacent to the putative 1,2-propanediol dehydratase in the BMC loci (Table 1). One possible role for the BMC is to protect the cell from the radical intermediate in this enzymatic reaction.

The resulting propionaldehyde can then be converted to either propanol by the alcohol dehydrogenase or to propionate by the combined action of the propionaldehyde dehydrogenase and phosphate propanoyltransferase (Fig. 6). In essence, the *C. phytofermentans* fucose and rhamnose dissimilation pathways are a concatenation of three separate pathways found in some enteric bacteria, one for the anaerobic conversion of fucose to 1,2-propanediol, one for the anaerobic conversion of rhamnose to 1,2-propanediol and one for the conversion of 1,2-propanediol to propionate and propanol [44]. Comparative genomic analysis suggests that the proposed pathway may be present in multiple clostridial species, and indicates the importance of future experimental validation of this pathway.

Surprisingly, genes hypothesized to be directly involved in the release of fucose and rhamnose from lignocellulose were not regulated in the same way as the fucose and rhamnose dissimilatory genes. Putative alpha-L-fucosidases and rhamnogalacturonases were expressed at similar levels on all three sugars. This may reflect either a difference in their regulation or an error in the annotation. Very few such enzymes have been characterized.

Another unexpected result was the induction of the expression of a wide variety of lignocellulolytic enzymes, including cellulases, chitinases, and mannanases during growth on fucose and rhamnose (Table 4). *C. phytofermentans* probably does not encounter high concentrations of free fucose and rhamnose in its natural environment. It is possible that the regulatory network structure of *C. phytofermentans* has not evolved to respond to fucose by suppressing the remainder of the organism's polysaccharide-degradative machinery. Alternatively, fucose and rhamnose may actually induce a general polysaccharide degradation response in *C. phytofermentans*. Future work will address whether fucose and rhamnose can act as signaling molecules that induce a broad degradative response.

## Supporting Information

File S1 **Primer names, sequences and conditions used for qPCR analysis.**
(DOC)
