# Supplementary material for: Involvement of a Bacterial Microcompartment in the Metabolism of Fucose and Rhamnose by Clostridium phytofermentans
Source: PLoS One. 2013 Jan 28;8(1):e54337. doi: 10.1371/journal.pone.0054337 (PMC3557285; doi:10.1371/journal.pone.0054337)
Supplement: File S1 — Primer names, sequences and conditions used for qPCR analysis. (DOC) [file pone.0054337.s001.doc]

**File S1. Primer names, sequences and conditions used for qPCR analysis.**

| **Predicted function** | **Gene** | **Sequence** | **Annealing temperature in Celsius** |
| --- | --- | --- | --- |
| Fucose isomerase | Cphy_3155 | TGGAATGCGAGTTGAGTCTG | 60 |
|  | Cphy_3155 | CATTTCTCCTTCGTCCATGC | 60.6 |
| Propanediol dehydratase | Cphy_1174 | TAGCGTTAATCCGTGCCTTC | 60.2 |
|  | Cphy_1174 | GCAACACGTACCACCAAATG | 59.9 |
| BMC shell protein ccmL/eutN | Cphy_1184 | AATGTAGGAGCCGGAATAGG | 58.3 |
|  | Cphy_1184 | TCAATCGGCGAGTTTTCTG | 60.5 |
| Rhamnose isomerase | Cphy_1147 | AGGCAAGAAACCCAGAAGAG | 58.5 |
|  | Cphy_1147 | TCACGATCTGCAAACTCACC | 59.8 |
